# Normal variance in emphysema index measurements in 64 multidetector‐row computed tomography

**DOI:** 10.1120/jacmp.v14i4.4215

**Published:** 2013-07-08

**Authors:** Bruno Hochhegger, Klaus L. Irion, Giordano R.T. Alves, Arthur S. Souza, John Holemans, Dhivya Murthy, Edson Marchiori

**Affiliations:** ^1^ Radiology Division Federal University of Rio de Janeiro Rio de Janeiro Brazil; ^2^ Radiology Division The Royal Liverpool and Broadgreen University Hospitals Liverpool United Kingdom; ^3^ Radiology Division University Hospital of Santa Maria Santa Maria Brazil; ^4^ Radiology Division University of São José do Rio Preto São José do Rio Preto Brazil

**Keywords:** computed tomography, densitovolumetry, emphysema index

## Abstract

The purpose of this study was to identify the normal variance of emphysema index (EI) measured in examinations acquired with 64 multidetector‐row computed tomography (64‐MDCT). A longitudinal, noninterventional study was performed retrieving all patients in our institution who are currently registered in our lung nodule protocol. All patients with clinical, functional, or significant radiological changes were excluded. We assumed that EI should remain unchanged within a short period of time. We reviewed 475 MDCTs in order to select 50 clinically stable patients who had two sequential chest MDCTs performed within a time interval of less than three months, and who presented at least one lung free of abnormalities but emphysema. CT densitovolumetry was used to calculate EI with thresholds set at −950 Hounsfield units (HUs) (EI‐950) and −970 HUs (EI‐970); on both studies from each patient. We observed the variation of total lung volume (TLV), mean lung density (MDL), and EI for measurements at the baseline and at follow‐up scans. Differences observed between baseline and follow‐up measurements were: TLVμ=149ml; IC=μ+1.96(133); EI−950=0.02%; p95=0.89%; EI−970μ=0.04%; p95=0.23% and MLDμ=15HU; IC=μ+1.96(18). The correlations obtained were the following: TLV r=0.96, EI−950r=0.79, EI−970r=0.85. Accepting that emphysema would remain unchanged within three months on stable patients, differences of less than 0.89% for EI‐950 and of less than 0.23% for EI‐970 are within the variance of the method.

PACS number: 87.50.ct

## I. INTRODUCTION

Pulmonary emphysema is defined as an abnormal permanent enlargement of the airspaces distal to the terminal bronchioles, accompanied by destruction of their walls, without obvious fibrosis.^(1)^ This major public health threat is currently ranked 12th as a worldwide burden of disease and is projected to be ranked fifth by the year of 2020 as a cause of loss of quantity and quality of life.^(2)^ Computed tomography (CT) is currently the method of choice for noninvasive assessment of the anatomical changes caused by emphysema, since high correlation has been demonstrated between CT, histopathology diagnosis, and grading of emphysema.^(3)^

Modern multidetector CT (MDCT) technology allows the scanning of the whole lung using high‐resolution parameters (<1mm slice thickness) in a single scanning acquisition, which generally lasts less than 10 seconds. Therefore, even patients with recognized lung diseases, such as emphysema, often tolerate to hold the breath during this acquisition time. For now, MDCT scanners are widely available and the majority of them are marketed with standard postprocessing software, which includes tools capable of performing volumetric emphysema quantification based on the lung densities (CT densitovolumetry).

The accurate diagnosis, quantification, and assessment of the progression of emphysema are important tools in the investigation and management of this irreversible lung disease.^3^, ^4^, ^5^, ^6^ The treatment of incapacitating emphysema by lung volume reduction surgery (LVRS) requires knowledge of the pattern, distribution, and severity of the emphysema in order to predict the surgical outcome. CT plays an important role in the investigation of these patients and also in the investigation of patients under treatments for α1‐antitrypsin deficiency and other congenital conditions.^3^, ^4^, ^5^, ^6^, ^7^ CT densitovolumetry is also recommended for longitudinal studies of emphysema,^(3)^ for assessment of lung volume compatibility in lung transplantation, and for emphysematous patients who are about to be submitted to minimally invasive treatment such as endoscopic implantation of endobronchial valves or stents.^3^, ^4^, ^5^, ^6^, ^7^, ^8^ Previous studies have tested the variation of CT quantification of emphysema.^9^, ^10^, ^11^, ^12^, ^13^ However, the extrapolation of their results to the general population may not be valid, as they were either focused on selected groups of diseases or have utilized noncommercial software that are not diffused, and therefore the data are not generalizable. We selected patients with a small emphysema index (EI) because previous studies indicated greater variations in this group.^9^, ^10^

The aim of our study was to identify the variation of EI measured by CT densitovolumetry, using standard CT software in a 64 detector‐row CT scanner, in patients with lower than 6% of EI (without massive emphysema).

## II. MATERIALS AND METHODS

### A. Patients

The institutional board approved this retrospective, observational, and noninterventional study. Informed consent was waived for all participants. We retrospectively reviewed a cohort of 475 patients who underwent sequential MDCT for surveillance of a lung nodule, between July 2011 and July 2012. The measurements were done in the opposite lung (the one which did not contain the nodule under surveillance). We included all patients that followed the lung nodule protocol in our institution. All patients included had baseline and follow‐up scans acquired on the same MDCT scanner with identical acquisition and reconstruction protocols. The time interval between baseline and follow‐up scans was three months or less. In addition, only the examinations performed without intravenous contrast medium injection and with a 512×512 image matrix were included. Exclusion criteria included the presence of consolidation, ground‐glass pattern, atelectasis, pleural effusion, respiratory movement artifacts or other lung abnormalities that could alter the density of the lung assessed. We also excluded all patients with an emphysema index above 6%, measured using the threshold of −950 Hounsfield units (HUs). The final sample included 47 patients, 25 male and 22 female, totaling 94 MDCT studies.

### B. CT scans

Examinations were acquired using a Philips Brilliance‐64 scanner (Philips Medical Systems, Cleveland, Ohio) using a 64×0.625mm detector configuration. Parameters had been set to 48 mm collimation, table speed of 67.2 mm per tube rotation (pitch 1.4), 120 k V, and 200 mAs. The raw data were processed to create a set of 1 mm thick axial images with edge enhancing algorithm at 10 mm interval (which was used only for visual analysis to assess exclusion criteria), and a second set of images with continuous slices with thickness of 0.75 mm, with a standard reconstruction algorithm (without edge enhancing). The CT scanner was periodically calibrated, as recommended by the manufacturer, despite the absence of major service events during the study interval. All images were analyzed and processed by two thoracic radiologists, each one with more than 15 years of experience in chest CT.

### C. Emphysema quantification

Quantification of EI in the selected lung was obtained by CT densitovolumetry performed by postprocessing of the second set of images, using an Advantage Workstation (GE HealthCare, Milwaukee, WI), in the following sequence: 1) segmentation by density band (threshold between −1023HU and −250HU); 2) segmentation by object selection (keep object tool); 3) exclusion of the lung containing the nodule by demarcation of the region of interest (ROI) (we selected the contralateral lung to the nodule, as the lesion could affect the results^(14)^); 4) confirmation of the adequacy of the segmentation process by visual analysis of a 3D model of the remaining data created by volume rendering process (visualization curve set for demonstration of voxels with density between ‐1024 HU and +1024HU); 5) measurement of the volume of the selected lung (total lung volume (TLV) and the mean lung density (MLD)) using the histogram tool; and 6) measurement of the volume of “emphysema” by threshold band selection, setting the upper limit of the threshold band at −950HU (for EI‐950) and at −970HU (for EI‐970). The histogram tool also reported the percentage of “emphysema” (emphysema index) for each of these threshold bands.^15^, ^16^

**Figure 1:**
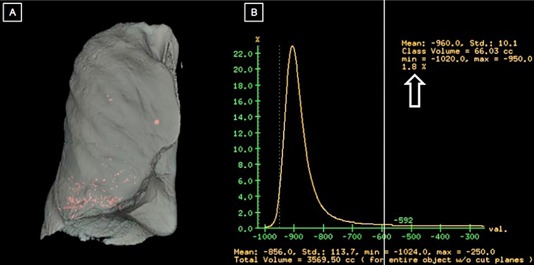
CT densitovolumetry: (a) 3D volume rendered CT shows the right total lung volume in white and the emphysematous volumes in red; (b) histogram of lung densities shows the emphysema index (large white arrow) and total lung volume.

The distribution of the emphysematous areas within the lung was analyzed by fusing both 3D virtual reality images created by volume rendering process: 1) image containing the whole selected lung with threshold band set to show all voxels with attenuation values between −1024HU and −250HU, with 2) image showing only the areas of emphysema within that lung [threshold band set at −1024HU and −970HU (for EI‐970) or set at −1024HU to −950HU (for EI‐950)], as shown in Fig. 1. The same process was repeated in the follow‐up study for each patient.

### D. Statistical analysis

Statistical analysis was performed using the software Microsoft EXCEL 5.0 (Microsoft, Redmond, WA) and the software MedCalc, version 8.1.1 (MedCalc Software, Mariakerke, Belgium). The presence of normal distribution was assessed with Kolmogorov‐Smirnov test. The tool “Normal Plot” provided a graphic representation of the data distribution. The variances of the parameters that showed a normal distribution were measured using mean and standard deviation. Percentiles were used to describe the variance of the parameters that did not present a normal distribution.

Differences between measurements from baseline scans (CT1) and follow‐up scans (CT2) were plotted against the mean, according to the method described by both Bland and Altman, and Camargo et al.^(9)^ Mean differences from CT1 and CT2 measurements were tested applying two‐tailed paired Student's t‐test. Correlation was measured using Pearson's formula. We also measured the intraclass correlations to identify possible differences amongst individuals.

## III. RESULTS

The results presented below reflect the acquisition parameters from normal dose CT examinations. This may be appropriate, if detecting the progression of emphysema is secondary to other clinical indications for requiring multiple CT scans. For low‐dose examinations (screening purposes only), one should consider data previously published.^(9)^ For instance, it has been demonstrated that emphysema quantification is affected by both mAs and kVp settings.^(17)^

From 50 clinically stable patients selected who had two subsequent chest MDCTs done within a time interval of less than three months and who presented with one lung free of abnormalities (other than emphysema), we excluded three outliers with EI−950>6%. Because of this, our final cohort consisted of 47 patients (25 male, 22 female), with mean age of 70 years (SD=9.2yrs). The mean time interval between baseline and follow‐up scan was 78 days (ranging from 68 to 90 days). Considering possible variances in TLV, MLD, and EI between CT1 and CT2 acquisitions, we observed significant differences only in EI registrations. The differences between such measurements are summarized in Table 1. Moreover, low correlation (r=0.2 for −950HU and r=0.1 for −970HU) was found between TLV and EI for all proposed thresholds. The correlations between CT1 and CT2 measurements are summarized in Table 2.

**Figure 2:**
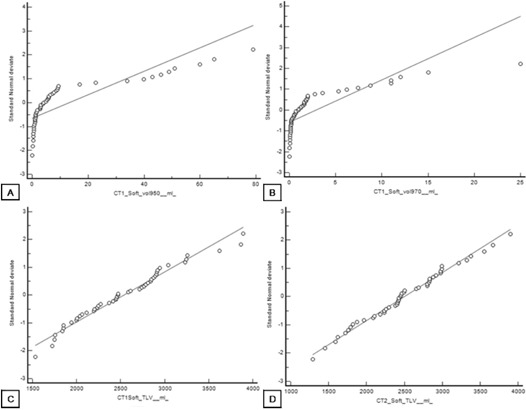
The normal plot showing the emphysema volumes in horizontal axis and the vertical axis gives the relative frequency in terms of the number of standard deviations from the mean. A straight reference line represents the normal distribution. If the sample data are near a normal distribution, the data points will be near this straight line. Volume of emphysema in CT1 (a); volume of emphysema in CT2 (b); total lung volume in CT1 (c); total lung volume in CT2 (d).

**Figure 3:**
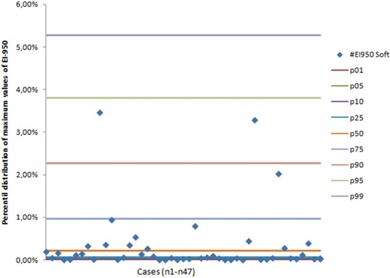
The differences in emphysema volumes (Dif EI=(EI−950CT1−EI−950CT2) against the percentile distribution of maximum EI measured in CT1 and CT2.

**Figure 4:**
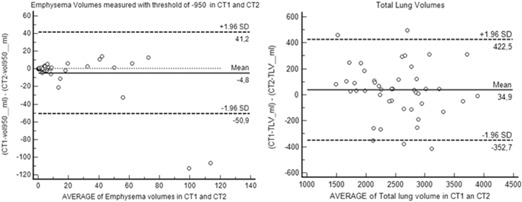
Bland‐Altman plots for EIs at (a) −950HU and (b) total lung volume. The x‐axes show the means of EIs (a) or TLV (b) on the baseline and repeat scans; the y‐axes show variable on the baseline scan subtracted from the repeat scan. The mean differences are shown with a solid line; the limits of agreement are shown with dashed lines. In (a), one increase in EI above the upper limit of agreement or a decrease below the lower limit of agreement has 95% likelihood to be a real progression or regression of emphysema.

Figure 2 shows that TLV had a normal distribution and that the distribution of EI was not normal in our sample. Figure 3 plots the differences in emphysema volumes (Dif EI=(EI−950CT1–EI−950CT2)) against the percentile distribution of maximum EI measured. Finally, Figure 4 shows the Bland‐Altman plots.

**Table 1:** Differences in measurements between CT1 and CT2

	*Dif TLV (ml)*	*Dif MLD (HU)*	Dif EI−970(%)	Per Dif EI−970(%)	Dif EI−950(%)	Per Dif EI−950(%)
Min	1	0	0.00%	0%	0.00%	103%
Max	494	58	0.60%	137%	3.45%	0%
Average	149	18	0.06%	20%	0.28%	20%
SD	133	15	0.13%		0.7%	
IC 95% Max	410	47	0.31%	93%	1.63%	75%
Median	102	15	0.02%	0%	0.04%	0%
Perc95	403	45	0.23%	150%	0.89%	125%

Dif TLV= differences in total lung Volumes between CT1 and CT2.

Dif MLD= differences in mean lung density between CT1 and CT2.

Dif EI−970= differences in emphysema indexes measured with a threshold of −970HU, between CT1 and CT2.

Dif EI−950= differences in emphysema indexes measured with a threshold of −950HU, between CT1 and CT2.

Dif EI−970(%)= percent of difference in emphysema indexes measured with a threshold of −970HU, between

CT1 and CT2, when comparison with maximum value of EI in CT1 and CT2.

Per Dif EI−950(%)= percent of maximum differences in emphysema indexes measured with a threshold of −950HU, between CT1 and CT2, when comparison with maximum value of EI in CT1 and CT2.

**Table 2:** Correlations between measurements

	TLV CT1×CT2	EI−950CT1×CT2	EI−970CT1×CT2	CT1EI−950×EI−970	CT2EI−950×EI−970
Correlation	r=0.94(0.89to0.96) ^a^	r=0.77(0.62to0.86) ^a^	r=0.76(0.61to0.86) ^a^	r=0.92(0.87to0.95) ^a^	r=0.84(0.73to0.91) ^a^
Intraclass Correlation	r=0.96(0.95to0.98) ^a^	r=0.79(0.64to0.89) ^a^	r=0.85(0.74to0.92) ^a^	r=0.5(0.07to0.73) ^a^	r=0.4(−0.03to0.65) ^a^

a Range

## IV. DISCUSSION

Variation of EI measurements has been tested for low‐dose CT,^9^, ^11^ for CT examinations acquired with thick slices,^10^, ^12^ and with a cardiac specific protocol.^(13)^ Previous publications have also shown that EI is influenced by CT acquisition parameters, including dose,^14^, ^16^ slice thickness, and scanner model.^14^, ^17^ Our study was designed to observe the variance of EI measurements on routine chest CT examinations performed without intravenous contrast medium using a 64‐row multidetector CT scanner, using standard radiation dose and thin collimation. The results may allow us to include a confidence interval when quantifying emphysema in patients with CT examinations that have not been specifically requested with this intent. In the practical scenario of routine CT examinations, without respiratory gating or other nonroutine procedures, we expected a larger variation when compared to the variance measured in ideal circumstances, using nonroutine techniques. Lower variance and higher correlation would probably be achievable if we had controlled the respiration to acquire both exams at the exact same degree of inspiration, if the interval between the scans was shorter (within few minutes or hours), or if we had controlled the patient for changes in body weight and hydration. However, these results would not reflect a real clinical scenario, thereby straying from our objective. Variation could also reflect interscan recalibration, degree of inspiration achieved by the patient, cardiac movement artifacts, positioning of the patient, and many other situations.

However, it is important to consider that our results are applicable only for patients with less than 6% of EI‐950 in any of both measurements. The value of 6% reflects the variance observed of EI in our cohort, after removing three statistical outliers. Moreover, previous studies showed greater variation in groups with lower EI.^9^, ^11^ Considering that the distribution of EI observed in our cohort was not normal, we defined the confidence interval using percentiles instead of mean and standard deviation. We accepted an α‐error of 5%, thus informing the 95th percentile, but we also included the 90th percentile in our tables.

Our results demonstrated that the maximum variation in EI‐950 was 0.89% (95th percentile) and 0.23% for EI‐970 (95th percentile). Therefore, in routine nonenhanced MDCT examinations, only variations above these values may represent progression of the disease. When we compare our data with previous studies that performed low‐dose examinations,^(9)^ we found a similar variance (1.1% for EI‐950). As suggested by other authors, these small differences could reflect the severity of emphysema of both cohorts and also the different radiation dose.^18^, ^19^ When we compare the maximum variation of EI with the mean of EI in CT1 and CT2, it was observed that the variation of emphysema index measured using the threshold at −950HU can be up to 125% at the 95th percentile. For measurements with threshold set at −970HU, variation can reach 150% at the 95th percentile.

Rosenblum et al.^20^, ^21^ were the first to use lung density to measure pulmonary emphysema on CT exams. Since their initial reports, various publications have suggested thresholds for distinguishing between normal and emphysematous lungs. Good correlation with histopathology grading was shown for the threshold −910HU for exams performed with thick slices and with the use of intravenous contrast medium.^(22)^ Gevenois et al.^(15)^ suggested the threshold −950HU, which has been used by various subsequent studies. The threshold −950HU has been studied in volumetric quantification of emphysema using helical CT in healthy nonsmokers, and it is known that an EI−950<0.35% should be regarded within a normal range.^(23)^ Recently, Madani et al.^(16)^ reported a stronger correlation between CT and histopathology grading for the threshold set at −970HU, as compared with the threshold at −950HU. It is important to consider, however, that despite using a MDCT scanner, results of the Madani study were based on individual axial images and not on volumetric analysis. Therefore, there is no consensus until now about the best threshold for the volumetric analysis of emphysema in MDCT scanners.

CT attenuation values, or densities, are measured in HU. The Hounsfield scale was created attributing −1000HU to the density of air and 0 HU to the density of water. For example, a mean attenuation value of −600HU measured in a ROI within the lung contains, on average, 60% air and 40% of soft tissue (or lung parenchyma).^(24)^ Different degrees of inspiration will therefore influence the results.^23^, ^24^ In this context, the MLD can vary under the influence of the degree of inspiration, the presence of interstitial lung disease, the use of intravenous contrast medium, and other conditions.^(25)^ On the other hand, it has been shown that there is an excellent agreement between EI measured in CT exams acquired at inspiration without spirometric control with EI measured on CT exams controlled by spirometric volume gating at 90% of the vital capacity. This leads to the conclusion that the lack of volume spirometric control does not appear to adversely affect EI results, at least for patients with extensive emphysema (≍ 50%), measured with threshold set at −900HU.^(12)^

Shaker et al.^(10)^ reported a high correlation between EI and TLV with higher values of EI. In contrast, we found a low correlation between EI and TLV when considering patients with EI<6% and variations of TLV in the range of 149ml±133ml. Similar findings were described by Gietema et al.^(9)^ Stoel et al.^(18)^ reported the importance of adjusting EI scores accordingly to TLV variation, especially when TLV differences larger than 200 ml are observed between baseline and follow‐up scans. We have not used such adjustments, since we have not found significant correlation between the variation of EI and the variation of TLV in our cohort (r=0.2). Additionally, only about 25% of our patients have TLV variation larger than 200 ml.

Low‐dose protocol has been recommended and tested for investigations performed with the only purpose of quantifying extensive emphysema.^10^, ^26^ However, low dose may be not sensitive to less severe disease because of the increased image noise in low‐dose CT, which can be reduced, but not eliminated, when applying a noise reduction filter.^(27)^ Moreover, it is important to have reference values for quantifications performed on standard dose examinations, as it would be inappropriate to repeat a CT just with this intent in patients who already have a CT chest done to address other clinical questions. Our results allow us to confirm stability or progression of emphysema in patients with previous CT examinations. The clinical significance of quantitative measurements is under study and it cannot progress without reference values of normality and confidence intervals of the measurements.

Percentile density (PD) has been proposed for longitudinal studies of emphysema.^(28)^ PD is defined as the attenuation value below which a certain percentage (usually 15%) of the voxels are, based on the frequency distribution histogram. The 15th percentile has been suggested for evaluation of emphysema progression.^(3)^ Madani et al.,^(16)^ however, found a better correlation between the 1st percentile and histopathology (r=0.57), when compared with the same correlation for the 15th percentile (r=0.5). The same authors also observed a correlation of r=0.58 between EI‐950 and histopathology, and also between EI‐970 and histopathology. While showing poorer results, we agree that PD can also be used for quantification of emphysema. However, unlike PD, we believe that EI directly reflects the phenomenon of destruction (or elimination) of lung tissue that occurs in emphysema, therefore diminishing the partial effect of air and lung structures on each voxel of the affected zone.

Our study also has some obvious limitations. First, it is focused on patients with EI‐950 below 6%. Therefore, our results cannot to be extrapolated to patients with more severe emphysema. Also, our results cannot be validated to CT scans performed with intravenous contrast medium. The cutoff effect at 0% probably did not represent bias in our cohort, as only one of our patients had EI−950=0% at both baseline and follow‐up scans.^(29)^ The assumption that EI would remain stable within the interval of three months could be a limitation, since it could be unrealistic. However, considering emphysema is a partially irreversible disease, the effect of this limitation is less relevant.

## V. CONCLUSIONS

For examinations acquired in 64‐row multidetector CT scanners, with a standard radiation dose and without intravenous contrast medium, only differences larger than 0.89% (assuming the 95th percentile as a confidence interval) should be considered as an actual progression of pulmonary emphysema, for a threshold set at −950HU. For the same conditions, but with the threshold set at −970HU, differences above 0.23% for the 95th percentile should be considered as real disease progression.

## Supporting information

Supplementary Material
