# Supplementary material for: Normal variance in emphysema index measurements in 64 multidetector‐row computed tomography
Source: J Appl Clin Med Phys. 2013 Jul 8;14(4):254–62. doi: 10.1120/jacmp.v14i4.4215 (PMC5714530; doi:10.1120/jacmp.v14i4.4215)
Supplement: Supplementary file 1 — Supplementary Material [file ACM2-14-254-s001.doc]

Normal variance in emphysema index measurements in 64 multi detector-row computed tomography

Short Title: Variation of emphysema index measurements in 64-MDCT

**Bruno Hochhegger, MD (1)**

**Klaus Irion, MD, PhD, FRCR (2)**

**Edson Marchiori, MD, PhD (1, 3)**

**Giordano R T Alves, MD (4)**

**Arthur S Souza Jr, MD, PhD (5)**

**John Holemans, MD, FRCR (2)**

**Dhivya Murthy, MD (2)**

*(1). Rio de Janeiro Federal University, Rio de Janeiro, Brazil.*

*(2). Liverpool heart and chest Hospital, Liverpool, United Kingdom.*

*(3). Fluminense Federal University, Rio de Janeiro, Brazil.*

*(4). Federal University of Santa Maria, Santa Maria, Brazil.*

*(5). University of São José do Rio Preto, São José do Rio Preto, Brazil.*

Conducted at: Rio de Janeiro Federal University, Rio de Janeiro, Brazil.

***Corresponding author:***

Giordano R T Alves, MD

Federal University of Santa Maria,

University Hospital of Santa Maria, Radiology Division,

Roraima Avenue, 1000, 97105-900.

Santa Maria, Rio Grande do Sul State, Brazil.

+555599159009

[*grtalves@gmail.com*](mailto:grtalves@gmail.com)
